# The complete mitochondrial genome of *Parnassius mercurius* Grum-Grshimailo (Lepidoptera: Papilionidae: Parnassiinae)

**DOI:** 10.1080/23802359.2019.1710279

**Published:** 2020-01-14

**Authors:** Chengcai Si, Keke Chen, Jiasheng Hao

**Affiliations:** Laboratory of Molecular Evolution and Biodiversity, College of Life Sciences, Anhui Normal University, Wuhu, Anhui, P. R. China

**Keywords:** Mitochondrial genome, phylogeny, Papilionidae, Parnassiinae, *Parnassius mercurius*

## Abstract

The mitogenome of *Parnassius mercurius* Grum-Grshimailo is determined to be 15,372 bp in length, including 37 typical insect mitochondrial genes and an AT-rich region. All PCGs start with ATN, except for *COI* with CGA; 12 of 13 PCGs harbour the common stop codon TAA or TAG, whereas *COII* end with a single T. The *lrRNA* and *srRNA* genes are 1344 bp and 775 bp in length, respectively. The AT-rich region contains several features characteristic of the lepidopterans. Phylogenetic analysis shows that *P. mercurius* is the closest relative of *P. epaphus* and *P. nomion* lineage, rather than the *P. jacquemontii*.

The *Parnassius mercurius* Grum-Grshimailo belong to the genus *Parnassius* (Lepidoptera: Papilionidae: Parnassiinae), and all species of this genus are mainly distributed in the mountainous areas of central Asia, the Himalayas and western China (Katoh et al. 2005; Omoto et al. 2009). According to morphological characteristics, *P. mercurius* was once considered a subspecies of *Parnassius jacquemontii* (Chou 1999), however, it was now considered an effective species based on mitochondrial DNA fragments (Michel et al. 2008).

In recent decades, insect mitogenomes have been widely used in studies of phylogenetic relationships, phylogeography and population genetics, etc. (Wang et al. 2015). In this study, we newly sequenced and characterized the complete mitogenome of the *P. mercurius*, meanwhile, we conducted the phylogenetic analysis of the species with other related *Parnassius* species. *P. mercurius* was collected from Menyuan County (E101.62, N37.37), Qinghai Province, China in July 2018. A voucher specimen (ANUH-20180725) was kept in the Herbarium of Anhui Normal University, Wuhu, China. Genomic DNA was extracted from the thorax tissues using Sangon Animal Genomic DNA Isolation Kit (Shanghai, China) and the PCR amplification and sequencing were conducted after Chen et al. (2014). The resultant reads were assembled and annotated using the BioEdit 7.0.5 (Hall et al. 2011) and MEGA 7.0 (Kumar et al. 2016).

The complete mitogenome of *P. mercurius* is 15,372 bp in size (GenBank accession No. MN728989), containing 13 protein-coding genes (PCGs), 2 ribosomal RNA genes (rRNAs), 22 transfer RNA genes (tRNAs), and an AT-rich control region. The nucleotide compositions of the genome are significantly AT biased (81.4%). All PCGs are initiated by typical ATN codons, except the *COI*, which utilizes CGA as its start codon. Twelve PCGs use standard TAA or TAG as the termination codons, while the *COII* end with the incomplete termination codon T. This phenomenon of partial termination codons is observed in all sequenced lepidopteran insects (Kim et al. 2009). All tRNAs have the typical clover-leaf secondary structures except for *tRNA^Ser^* (AGN), as seen in all other determined butterfly species (Park et al. 2012). The *lrRNA* and *srRNA* genes are 1344 bp and 775 bp in length, respectively. The AT-rich region is 504 bp in size and contains several structures characteristic of lepidopterans, including the motif ATAGA followed by a 20 bp poly-T stretch, a 11 bp poly-A stretch and a microsatellite-like (TA)_8_ preceded by the ATTTA motif (Shi et al. 2018).

Using *Sericinus montela* and *Luehdorfia chinensis* as the outgroups, we performed a neighbor-joining (NJ) phylogenetic analysis with MEGA 7.0 (Kumar et al. 2016) based on the concatenated four mitochondrial DNA sequence data (*lrRNA*, *COI*, *ND1*, and *ND5*) to elucidate the phylogenetic relationship of *P. mercurius* with other *Parnassius* species. The resultant phylogenetic trees showed that the *Parnassius* species are distinctly divided into eight clades (subgenera), and the *P. mercurius* is the closest relative of *Parnassius epaphus* and *Parnassius nomion* within the subgenus *Parnassius* (clade VIII) (Figure 1). Therefore, *P. mercurius* should be taken as an effective species, rather than as a subspecies of *P. jacquemontii*.

**Figure 1.**
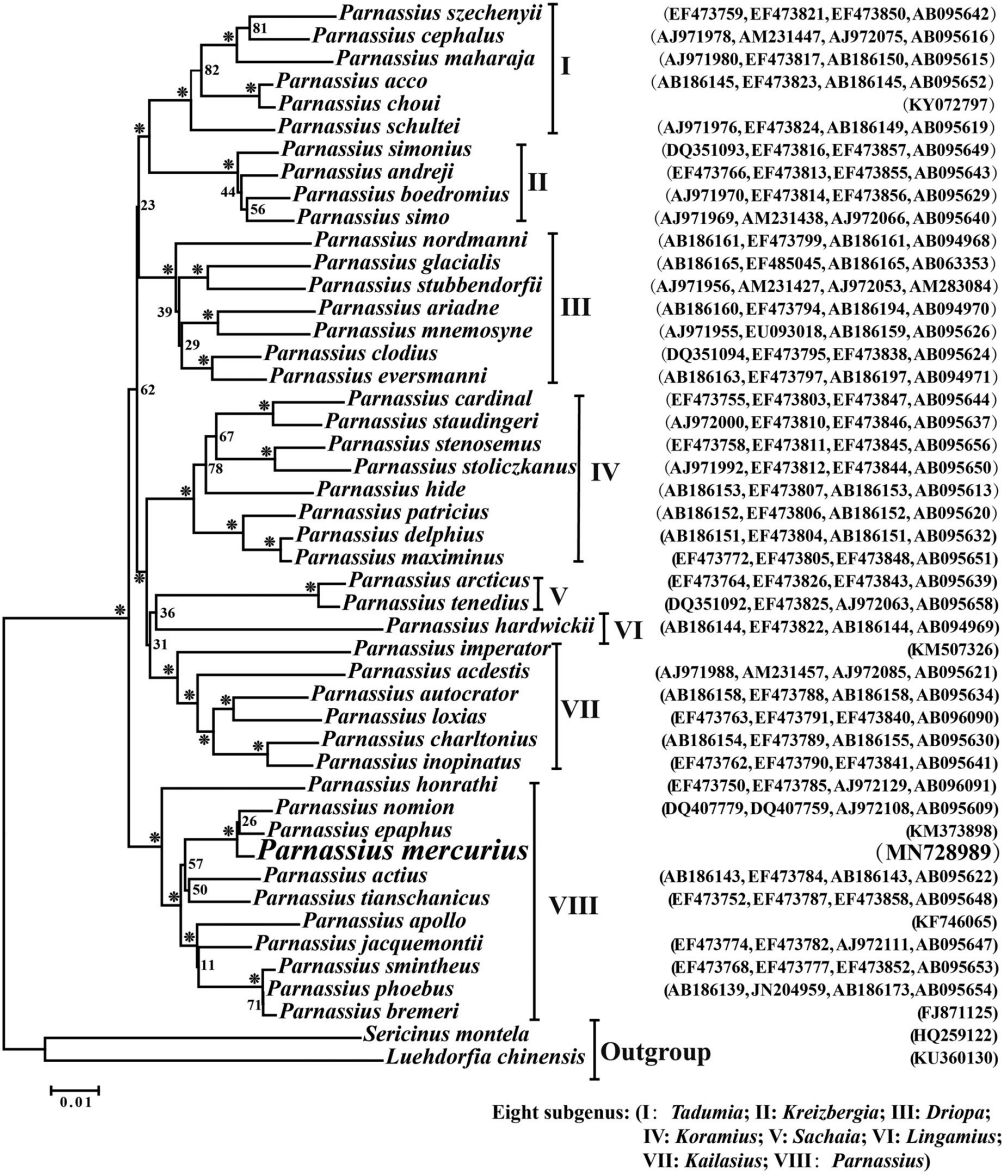
The neighbor-joining (NJ) phylogenetic tree of *P. mercurius* and other *Parnassius* species. Phylogenetic reconstruction was performed from a concatenated matrix of four mitochondrial genes (*lrRNA*, *COI*, *ND1*, and *ND5* genes). The numbers beside the nodes are percentages of 1000 bootstrap values (*≥85%). The alphanumeric characters in parentheses represent the GenBank accession numbers.
